# A Retrospective Patient Chart Review and Survey in Patients with Cryopyrin-associated Periodic Syndromes Treated with Anakinra

**DOI:** 10.36469/9860

**Published:** 2013-08-21

**Authors:** Helen J. Lachmann, Renée J.G. Arnold, Marco Gattorno, Isabelle Koné-Paut, Alberto Ferreira, Jasmin Kümmerle-Deschner

**Affiliations:** 1 UCL Medical School, London, UK; 2 Arnold Consultancy & Technology LLC, New York, NY USA; Mount Sinai School of Medicine, New York, NY USA; 3 UO Pediatria II, G. Gaslini Scientific Institute, Italy; 4 Pediatric Rheumatology, CEREMAI, Hôpital Kremlin Bicêtre, University of Paris SUD, France; 5 Novartis Pharma AG, Basel, Switzerland; 6 Division of Pediatric Rheumatology, Department of Pediatrics University Hospital Tübingen, Germany

**Keywords:** flares, perceptions, observational, retrospective, anakinra, caps, cryopyrin-associated periodic syndromes

## Abstract

**Background:** Cryopyrin-associated periodic syndromes (CAPS) is a group of rare autoinflammatory diseases. Little is known about the burden of disease, patients’ views on treatment, and adverse events (AEs) with current therapy.

**Objectives:** The main study objective was to quantify the patients’ burden of disease in terms of flares and resource use and to characterize patient symptomatology and tolerability of treatment with anakinra. A secondary objective included comparing chart review and patient recall of symptoms and AEs.

**Methods:** A retrospective medical chart review and concurrent online patient survey was conducted in four European countries. Data 12 months prior to initiation of/during anakinra treatment were entered into web-based case report forms by study groups.

**Results:** Forty-two patients received/were receiving anakinra as primary treatment for at least 12 months. Patients experienced a 79.5% reduction in flares after commencing anakinra treatment. During the past 12 months on anakinra, four of five (80%) patients who recalled experiencing flares reported cancelling social activities and staying home as the most common courses of action. Most common AEs were injection site pain upon treatment initiation and weight gain. According to patient recall, 12 of 21 patients (57.1%) discontinued anakinra to enter another clinical trial; of the 12, eight (38%) specifically discontinued anakinra only for that reason, and four patients cited entering a clinical trial as one of many reasons for discontinuing anakinra.

**Conclusions:** To our knowledge, this is the most comprehensive survey of patient experience with CAPS. Although improved, CAPS treatment remains suboptimal and a significant burden is placed upon patients, caregivers, and the healthcare system. With new agents available, it will be important to compare outcomes in patients using all therapeutic options.

## INTRODUCTION

Cryopyrin-associated periodic syndrome (CAPS) is a rare autoinflammatory disease which encompasses a disease severity spectrum from familial cold autoinflammatory syndrome (FCAS) to Muckle-Wells syndrome (MWS) and neonatal onset multisystem inflammatory disorder (NOMID).[1,2] The extreme rarity of CAPS, coupled with relatively low awareness among healthcare providers, often leads to misdiagnosis. The current estimated prevalence of FCAS is less than one in a million[3] (based on confirmed diagnoses of patients treated in specialized autoinflammatory disease centers), and it is assumed to be the same in MWS and NOMID.

The gene associated with CAPS is NLRP3, a constituent of the caspase 1 inflammasome. Mutations in NLRP3 result in constitutive overactivation of the caspase 1 inflammasome, causing excess production of interleukin-1ß (IL-1ß), a key pro-inflammatory cytokine. The role of IL-1 in CAPS became evident when anakinra, given by subcutaneous injection, was used empirically to treat patients[4] and revolutionised treatment. Until 2009, anakinra (Kineret®, Swedish Orphan Biovitrum), used off-label, was the only anti-IL1 agent widely available to patients. Anakinra was approved by the European Commission in 2002 for the treatment of rheumatoid arthritis in combination with methotrexate for patients with an inadequate response to methotrexate alone. Though approved by the European commission, there is limited information regarding certain aspects of treatment with anakinra. For instance, anakinra injection site reactions in children have not been observed in depth. Additionally, treatment patterns (including dosing) have not yet been well characterised for anakinra in CAPS patients due to the small size of the population and the limited experience with anakinra treatment.

In the absence of approved medicines, anakinra has been used to manage CAPS symptoms;[4-10] however, expert opinion and sparse data suggest that the treatment of CAPS was suboptimal in some patients and compliance may have been limited. For example, refusal of daily treatment was observed in a recent study.[8] Poorly treated or untreated CAPS leads to considerable morbidity and impacts education, social relationships, and occupation.[10] The objective of the current study was to quantify the burden of disease on CAPS patients and to characterize patient symptomatology and tolerability of treatment with anakinra. A secondary objective of the study included comparing chart review and patient recall of symptoms and AEs. Due to the subjectivity of CAPS symptoms and the suboptimal treatment many patients receive, it was determined that many patients may not be as forthcoming with physicians regarding their symptoms and AEs. Therefore, it was imperative that we assess patient perception and compare it to site records.

## PATIENTS AND METHODS

### Study Design

This was a retrospective patient chart review and survey of CAPS patients. The study took place at four centres of excellence—one each in the United Kingdom, France, Italy and Germany.

### Participants

The study's inclusion criteria were: genotypic and phenotypic diagnosis of CAPS; under continuous care of a physician at one of the study sites specifically for CAPS for at least 12 months; and at least a 12-month treatment period not participating in a clinical study with IL-1 inhibitors. The only exclusion was treatment with IL-1 inhibitors associated with a clinical study.

### Data Source and Measurement

After Ethics Committee approval in each country, sites recruited patients and obtained informed consent from patients. Data were directly collected from patients during the period June 2009 - August 2009. No patient-specific information that would allow patient identification was recorded. Data were entered onto case report forms by data managers and principal investigators at the sites and by patients remotely.

Site record data collection was completed through a chart review over the 12 months prior to patients completing the online surveys. Patients were first asked to recall flares over the previous month on anakinra treatment. The patients individually determined the definition of a flare, generally, in terms of any clinical disease activity due to CAPS and each period of activity counted as a flare. Thus, a patient with symptoms every evening would have 30 flares a month. If patients did not recall having any flares during the last month, they were asked to recall flares over the previous 12 months. Sites were asked to consider the number of flares per patient over the previous 12 months; these data were normalised to flares per month.

## RESULTS

### Descriptive Data

Fifty Caucasian patients (32 adults and 18 children) were enrolled from four Centres of Excellence for CAPS. The United Kingdom, Germany, Italy, and France provided 18, 15, 11, and 6 patients, respectively. All 50 patients who were contacted agreed to participate in the study. Patients had a mean age of 29.0 (standard deviation [SD] 17.5) years and had been diagnosed with CAPS for a mean of 4.3 (SD 3.2) years with the following phenotypes: FCAS/familial cold urticaria syndrome (FCUS) (7), FCAS/FCUS–MWS overlap (1), MWS (32), MWS-NOMID/chronic infantile neurological, cutaneous and articular (CINCA) syndrome overlap (3) and NOMID/CINCA (7) (see Table 1).

**Table 1.** Patient Demographics

| Total Enrolled Patients, N (%), N=50 | |
|---|---|
| **Gender** | |
| Male | 21 (42%) |
| Female | 29 (58%) |
| **Age, years (mean, SD)** | 29.0 (17.5) |
| <18 | 18 (36%) |
| ≥18 | 32 (64%) |
| **Time since Diagnosis, Years (mean, SD)** | 4.3 (3.2) |
| **Body Weight (kg. mean, SD)** | 56.5 (24.3) |
| **Height (cm. mean, SD)** | 154.4 (21.2) |
| **Phenotype** | |
| FCAS/FCUS Only | 7 (14%) |
| Muckle-Wells Only | 32 (64%) |
| NOMID/CINCA Only | 7 (14%) |
| FCAS/FCUS Muckle-Wells Overlap | 1 (2%) |
| Mucle-Wells NOMID/CINCA Overlap | 3 (6%) |

SD=standard deviation; FCAS=familial cold autoinflammatory syndrome; FCUS=familial cold urticaria syndrome; NOMID=neonatal onset multisystem inflammatory disease; CINCA=chronic infantile neurological, cutaneous and articular syndrome

## Outcomes Data

*Primary CAPS Treatment*

Forty-two patients had either received or were receiving anakinra as a primary treatment for at least 12 months. Of these, 39 required daily injections; the other three received alternate day injections. According to patients, the mean duration of their treatment was 2.1 (SD 1.5) years. Patient dosage ranged from a mean minimum of 1.1 (SD 0.5) mg/kg to a mean maximum of 1.8 (SD 1.0) mg/kg. The other eight patients were prescribed or had taken colchicine, infliximab, prednisolone, thalidomide, paracetamol or no medication.

*Flare Characterization/Patient Outcomes*

According to site records, patients experienced a 79.5% reduction in flares, from a mean of 15.1 (SD 12.6) flares prior to anakinra treatment to a mean of 3.1 (SD 7.8) flares after commencing anakinra treatment (Table 2). Of the 41 patients that confirmed treatment with anakinra in the survey, 13 (32%) patients recalled that they had experienced flares during the month prior to completing their online questionnaires (Figure 1). As reported by these patients, the number of flares in the previous month while taking anakinra (regardless of flare severity) was an average of 2.7 (SD 4.2) per patient, not significantly different from the mean of 3.1 from chart review, but varied widely between patients from only one per month to daily frequency (Table 2). The severity of flares varied between individuals, and patients usually had more than one level of flare severity in the month.

**Table 2.** Comparison between Site Records and Patient Recall of Flares Prior to and during Anakinra Treatment

| | Site Records | | Patient Recall | |
|---|---|---|---|---|
| | **Number of Patients with Flares** | **Number of Flares per Month Mean (SD)** | **Number of Patients with Flares in Last Month** | **Number of Flares Last Month Mean (SD)** |
| **During Anakinra Use** | N=14 | 3.1 (7.8) | N=13 | 2.7 (4.2) |
| **Prior to Anakinra Use** | N=44 | 15.1 (12.6) | | N/A |

SD=standard deviation

The most common flare symptoms reported by patients included fatigue/low energy (92%), muscle or joint aches/pains (77%), headache (62%), fever/chills (54%), eye redness (46%), pain in extremities (39%), skin rash (39%), abdominal pain (23%) and edema in extremities (15%). Six of the 13 patients (46%) who experienced flares stayed at home when they felt unwell, and 31% either missed school or slept as a way to deal with their flares. Of those patients experiencing flares, the majority had flares that were mild (75%) or moderate (77%) in severity and 52% had severe flares for the 12 months back from the index date of anakinra initiation. For those five patients who reported flares during the past 12 months while on anakinra, four (80%) reported cancelling social activities and staying home as the most common courses of action.

**Figure 1.** Characterization of Enrolled Patient Population

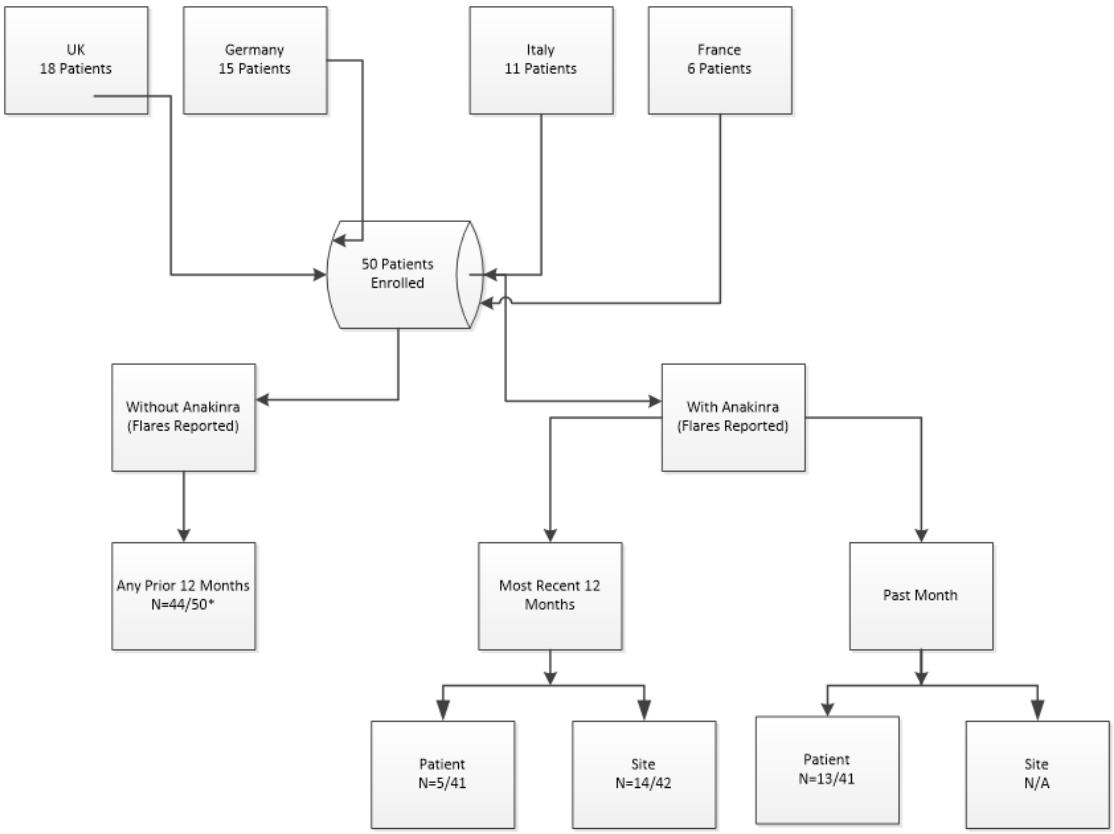

Three of the 5 (60%) respondents reported that they took pain medication. Resource use/course of action with anakinra over the past 12 months is shown in Table 3. According to site records, over the most recent 12 months on anakinra, patients with more severe disease tended to have office visits (one of four patients reporting severe flares had an office visit) and there was one hospitalization (again 25% of patients reporting severe flares). Those with less severe disease contacted the sites via telephone. No emergency room visits were seen.

**Table 3.** Total Course of Action over the Past 12 Months with Anakinra, According to Patient Recall

| Course of Action | Total, N=5 |
|---|---|
| Used Medication | 2 (40%) |
| Took Pain Medication | 3 (60%) |
| Stayed at Home | 4 (80%) |
| Missed School | 1 (20%) |
| Slept | 2 (40%) |
| Cancelled Social Activities | 4 (80%) |
| Telephone Call to MD | 1 (20%) |
| Office Visit | 2 (40%) |
| Hospitalization | 1 (20%) |

Few additional medications for the treatment of flares were reported in the site records for these patients; only 3% of patients reporting moderate flares were recorded as having been prescribed over the counter (OTC) pain medications. The topical therapy was only used as needed and, therefore, not previously reported as a concomitant medication. According to patient recall, they took pain medications (39%), either prescribed or non-prescription, stayed at home (46.2%) and slept (30.8%). Approximately one fourth of patients (23.1%) reported visiting a doctor.

*Anakinra Adverse Events and Discontinuations*

Common AEs from patient recall were pain (46.3%) and swelling (34.1%) at the injection site, followed by weight gain (29.3%). Patients also reported redness and itching at the injection site. In addition, 14 patients recalled having other reactions at the injection site. Twelve patients recollected no AEs whilst treated with anakinra for 12 months. A listing of AEs, while on anakinra, can be seen in Table 4.

**Table 4.** Anakinra Adverse Events throughout Data Collection Period: Comparison between Patient Recall and Site Records

| Adverse Event | Patient Recall N (%) | Site Records N (%) |
|---|---|---|
| Total | 41 (100.0%) | 42 (100.0%) |
| Data Collection Period (months) | The most current 12 months of being seen at the site prior to clinical study inclusion | 12 months pre-anakinra therapy initiation date and most recent 12 months of being seen at the site and pre-clinical study enrollment |
| None | 12 (29.3%) | 13 (31.0%) |
| Injection Site Pain upon Treatment Initiation | 19 (6.3%) | 21 (50.0%) |
| Injection Site Edema upon Treatment Initiation | 14 (34.1%) | 10 (23.8%) |
| Weight Gain | 12 (29.3%) | 15 (35.7%) |
| Injection Site other Reaction | 14 (34.1%) | 2 (4.8%) |
| Diarrhea | 1 (2.4%) | 1 (2.4%) |
| Fever | 0 (0.0%) | 1 (2.4%) |
| Rhinitis | 1 (2.4%) | 1 (2.4%) |
| Nasopharyngitis | 2 (4.9%) | 0 (0.0%) |
| Gastroenteritis | 1 (2.4%) | 0 (0.0%) |
| Influenza/Viral Infection | 1 (2.4%) | 1 (2.4%) |
| Urinary Tract Infection | 1 (2.4%) | 0 (0.0%) |
| Other Infections | 1 (2.4%) | 0 (0.0%) |
| Other: (Needle anxiety, Aphthous ulcers, Needle phobia) | 2 (4.9%) | 3 (7.1%) |

Weight gain in adults was noted as a substantial problem in both site records and by patients. Dizziness/vertigo, not alleviated by anakinra, was also particularly noted by patients. Symptoms of vertigo failed to improve following treatment with anakinra, having a 4% incidence. In addition, lack of effectiveness of treatment was cited by 10% of patients as a reason for discontinuing anakinra. There was insufficient evidence in the charts to determine any changes in delayed puberty, impaired growth, auditory loss, visual loss, impaired fertility, clubbing, lower limb edema, tinnitus, vertigo, learning difficulties and skeletal abnormalities.

## DISCUSSION

CAPS is a genetic disease which requires lifelong treatment to relieve symptoms and prevent long-term complications. The recognition that anti IL-1 treatment was effective in CAPS has transformed the patient experience. There is suggestive evidence that treatment can prevent progressive renal damage from AA amyloidosis,[11] while the effect on prevention or reversal of other long-term complications, such as learning difficulties that may have been present prior to the CAPS diagnosis, is yet to be seen. There have been no randomised studies of the efficacy or safety and tolerability profile of anakinra in the CAPS population. Efficacy has been shown through published single-case reports and case series.[4] All reports describe significant alleviation of CAPS-related symptoms and signs, with normalisation of biochemical markers of inflammation from daily injections. Studies evaluating the long-term efficacy and safety of anakinra in pediatric CAPS patients have suggested continued long-term efficacy based on limited exposure.[4,6] Local injection site reactions and pain have been the most frequently reported AEs in CAPS literature and potentially can seriously limit treatment acceptance, particularly in children.[6,12]

Of note, other drugs have been tried in the past and were discontinued due to lack of efficacy prior to the recognition that CAPS is IL-1-driven. Indeed, in the current study, colchicine was reported as a primary medication for one patient in France for 6 months in 2005 prior to anakinra prescription. Infliximab, prednisolone and thalidomide were used as primary medications by the United Kingdom site for one patient before 2002.

The current study examined a relatively large patient cohort and assessed symptoms, patient reaction to flares and tolerability to anakinra treatment. Although patients experienced a 79.5% reduction in flares after commencing anakinra treatment, greater than 30% of patients treated with anakinra recalled at least one flare, an average of 2.7 in the previous month, while on anakinra treatment. Lack of effectiveness cited by 10% of patients as a reason for discontinuing anakinra could have been due to non-adherence to the rigors of daily dosing or not adequately self-adjusting to the changing needs of the disease state. One could also interpret this as an improvement following treatment with anakinra, but not full relief from flares. Thus, as shown in this study, the current CAPS treatment is suboptimal in some respects, and a significant burden remains on patients, their caregivers and the healthcare system.

In an attempt to identify common symptoms and impact on the lives of CAPS patients, numerous studies have been carried out.[10,11,12] Patients have indicated that as burdensome symptoms continued to progress, work, school, family and social activities were significantly limited.[11] In the current study, patient recall over the previous month indicated that the burden of CAPS required greater than one third of patients to stay at home and sleep. For the majority of AEs, data from both patient recall and site records were in agreement. The difference noted between site records and patient recollection regarding the mean duration of treatment may have been due to patients self-adjusting their treatment. However, the temporal relationship between dose adjustments and symptom flares was not recorded.

Weight gain as an AE has not previously been mentioned in studies assessing the safety of anakinra, which suggests that this could be specific to CAPS patients. Studies have shown that IL-1Ra is an important regulator of adipogenesis, food intake and energy expenditure, and that it has both endocrine and paracrine effects on the hypothalamus and adipocytes, respectively, that may invoke weight gain.[13,14] Alternatively, it may be a result of complete control of inflammation and fever resulting in a reduction in energy expenditure and not be specifically related to anakinra. This would need to be confirmed in randomised clinical studies.

Sensorineural hearing loss is a well-recognised complication of CAPS. The effect of anakinra on hearing has been previously reported. In some cases treatment has been thought to have prevented further hearing loss.[15] Other publications have demonstrated no effect[16,17] on hearing loss from anakinra while several have shown at least some improvement.[18-22] In this study, impaired hearing was reported by 28 of the 50 patients (56.0%) prior to starting anakinra and 27 of 42 patients (54.0%) on anakinra, suggesting that established sensorineural hearing loss may not be reversible. This study was not designed to capture small changes in hearing, and treatment periods assessed could be as short as 1 year. So, the failure to detect a reduction in the number of patients reporting hearing loss does not mean that improvement did not or cannot occur.

There were clearly some limitations to the study. Recall bias was a problem, with fewer patients reporting flares over a 12-month period (N=5) than over the past month (N=13). Nonetheless, the comparison of site records and patient recall over the most recent 12 months while on anakinra suggests great similarity in perceptions between patients and their doctors. Furthermore, in a cohort study of an orphan disease, such as this one, according to Sackett, avoidance and/or measurement of recall bias is not possible.[23,24] The study may also have been subject to selection bias, but since everyone who fulfilled the inclusion criteria was approached, selection bias was minimized. Another potential study bias may have been due to the inclusion of only those patients who remained on the primary medication for 1 year. However, excluding the patients who withdrew after less than 1 year would have only introduced bias in favor of the medication.

Using a validated questionnaire for patient-reported outcomes would have been preferable, but these do not exist as yet, and the current study represents the first attempt to assess treatment, quantify flares and compare patient perception of symptoms or severity with those of physicians to estimate actual burden of disease on patients with CAPS. Another limitation of this study was that patients with different disease severities (FCAS to NOMID) were enrolled. Though enrolling patients with various disease severities was a necessity in order to get an appropriate population size, this may have had an impact on study outcomes.

Insufficient treatment response in some patients due to inadequate dosing may have been another limitation of this study. However, in the current study, both adults and children received 1.1 – 1.8 mg/kg/day, within the "therapeutic" range of 1-3 mg/kg/day reported for phenotype NOMID.[25] Dosages of 0.5-1.5mg/kg/d for FCAS and up to 3.5 mg/kg/d have also been recommended for MWS treatment; therefore, doses in the current study were generally within the low to middle of the recommended range.[26]

Lastly, since neither rilonacept nor canakinumab was available in Europe at the time of study data collection, we were unable to collect data on these agents.

## CONCLUSION

Cryopyrin-associated periodic syndrome (CAPS) is a rare autoinflammatory disease. The extreme rarity of CAPS, coupled with relatively low awareness among healthcare providers, often leads to misdiagnosis. Poorly treated or untreated CAPS can then lead to considerable morbidity and impact education, social relationships and occupation. Though existing agents have improved treatment, the current treatment of CAPS remains suboptimal in some respects, and a significant burden continues to be placed on patients, their caregivers and the healthcare system. With the availability of newly approved agents, such as rilonacept and canakinumab, it will be imperative to compare outcomes of patients using all therapeutic options.

## Acknowledgements

We gratefully acknowledge the sponsorship of Novartis Pharma AG in this research. We also acknowledge and greatly appreciate the expert assistance of the following individuals in Ethics Committee documentation and data collection: Thirusha Lane (UCL Medical School, UK), Signe Risom (UCL Medical School, UK), Isabelle Marie (Hôpital Kremlin Bicêtre, France), Sandra Hansmann (University Hospital Tübingen, Germany), Silvia Federici (G. Gaslini Scientific Institute, Italy) and Roberta Caorsi (G. Gaslini Scientific Institute, Italy). We would also like to thank Hal Hoffman (University of California at San Diego) and Philip Hawkins (UCL Medical School, UK) for their guidance and Sarah Evans (Arnold Consultancy & Technology LLC at the time of the study) for data collation and interpretation.

## Conflict of Interest Declaration

All except for AF have accepted payment from Arnold Consultancy & Technology LLC for the provision of patient data, interpretation of results. In terms of competing interests, MG received a speaker's fee from Novartis AG and SOBI and an educational grant from Novartis AG (for a Eurofever project); HL, IK, and JK received payment from Novartis AG for the following: being an investigator on studies, consultant; speaker's honoraria, and travel expenses. No other competing interest exists (including holding stocks or shares and/or patent applications).
